# Chromosome balanced translocation in newborn fetus founded during prenatal diagnosis: Three cases reports

**DOI:** 10.1097/MD.0000000000037345

**Published:** 2024-03-08

**Authors:** Lan Yao, Xun Kan, Yuxin Xia, Luyao Wang, Xueyu Zhao, Yingli Lu

**Affiliations:** a The Second Hospital of Jilin University, Changchun City, Jilin Province, China

**Keywords:** amniocentesis, case report, chromosomal balanced translocation, prenatal diagnosis

## Abstract

**Rationale:**

Because of the normal phenotype, carriers of specific chromosomal translocations are often diagnosed only after their development of associated malignancies, recurrent miscarriages, and reproductive difficulties. In this paper, we report primary balanced fetal chromosomal translocations by performing the necessary invasive prenatal diagnosis in couples with previous malformations coupled with prenatal testing suggesting a high risk for trisomy 21.

**Patient concerns:**

Case 1 and Case 2 couples had malformed children, and Case 3 couples had a high risk of trisomy 21 on noninvasive preconception serological testing.

**Diagnosis and intervention:**

A balanced chromosomal translocation diagnosis was confirmed by karyotyping of fetal cells obtained by amniocentesis.

**Outcomes:**

All 3 couples decided to continue their pregnancies after learning about the consequences of the chromosomal abnormalities. Approximately a year after the children were born, the staff of the Prenatal Diagnostic Center followed up with a phone call and found that the children physical development and intelligence were normal.

**Lesson:**

This case report reports healthy chromosomal balanced translocation newborns born to couples with poor maternal history and couples with abnormalities suggested by preconception testing, and followed up with the newborns to provide some experience in prenatal diagnosis and genetic counseling for chromosomal balanced translocations.

## 1. Introduction

Chromosomal abnormalities are a common cause of early spontaneous abortion and abnormal fetal development, and the source of fetal chromosomal abnormalities can be idiosyncratic or occur with one or both spouses. Chromosomal abnormalities are categorized into structural abnormalities (translocations, inversions, insertions, rearrangements, deletions, ring chromosomes, etc) and numerical abnormalities. Balanced chromosomal translocation occurs when 2 chromosomes break at one point and exchange their unattached segments with each other to form 2 newly derived chromosomes, which include both homologous and non-homologous chromosomes. The phenotype can be normal despite the chromosome segments’ owing to mutual translocation, which does not affect the total number of genes.^[1]^ Certain specific chromosomal balanced translocations are strongly associated with malignant tumors, especially those of the blood and lymphatic systems.^[2]^ Chromosomal translocation carriers in one of the couples are one of the causes of adverse pregnancies and deliveries.^[3]^ Because of their normal phenotype, some carriers of chromosomal translocations are usually diagnosed only after the onset of associated malignancies, recurrent miscarriages, or reproductive difficulties.

Reliable prenatal screening methods can identify pregnant women with fetal chromosomal abnormalities or congenital disabilities in the early and mid-pregnancy stages, helping expectant parents achieve better pregnancy outcomes and healthy offspring. According to maternal age, fetal nuchal translucency measurement, and maternal serum markers (most often accessible beta-hCG and PAPP-A), first-trimester screening calculates individual risks for trisomies 21, 18, and 13.^[4]^ The most effective noninvasive technique for determining trisomy 21, 18, and 13 risk at this time is cells-free fetal DNA. A meta-analysis showed that noninvasive DNA testing for trisomies 21, 13, and 18 in singleton pregnancies was 99.7%, 99.0%, and 97.9% with a false-positive rate of 0.04%, and 95.8% for monosomy X with a false-positive rate of 0.14%.^[5]^ For gravidas who have previously conceived an abnormal fetus and are suspected of conceiving an abnormal fetus by prenatal screening, direct fetal cell collection and accurate chromosomal analysis of the fetus are prerequisites. Currently, amniocentesis is the most widely used interventional prenatal diagnostic technique.^[6]^ Fetal cells or DNA can be obtained directly from amniotic fluid to analyze fetal karyotypes-, gene sequencing. Moreover, it can be used to determine whether fetal or intraamniotic infections are present using polymerase chain reaction or bacterial culture. Neural tube defects can be evaluated by measuring amniotic fluid alpha-fetoprotein and acetylcholinesterase to assess neural tube defects.^[7,8]^ Moreover, as a widely used prenatal diagnostic method, amniocentesis is relatively low-risk, and a systematic review and meta-analysis showed no significant risk of miscarriage increased by amniocentesis when women undergoing invasive prenatal testing and controls had a similar risk of chromosomal abnormalities.^[9]^ Furthermore, amniocentesis is safer after 14 to 15 weeks of gestation than before 14 to 15 weeks of gestation. A practice bulletin published in 2016 suggested that early amniocentesis (before 14 weeks gestation) is not recommended.^[6]^ An investigation comparing late amniocentesis (after 15 weeks of gestation) versus early amniocentesis (before 15 weeks gestation) in a randomized fashion and chorionic villus sampling (transabdominal or transvaginal) showed that late amniocentesis was safer than early amniocentesis.^[10]^

In this study, fetal cells obtained by amniocentesis were harvested, cultured, and sliced, and 20 phases of division were counted under an oil microscope. Five of them were analyzed by G-banding staining to obtain a report on chromosomal balanced translocation karyotype analysis of the fetus, this will offer some expertise in chromosomal balanced translocation genetic counseling and prenatal diagnostics.

## 2. Cases introductions

Informed consent was obtained from the patients to publish this case report and the accompanying images.

### 2.1. Case 1

**Figure 1.**
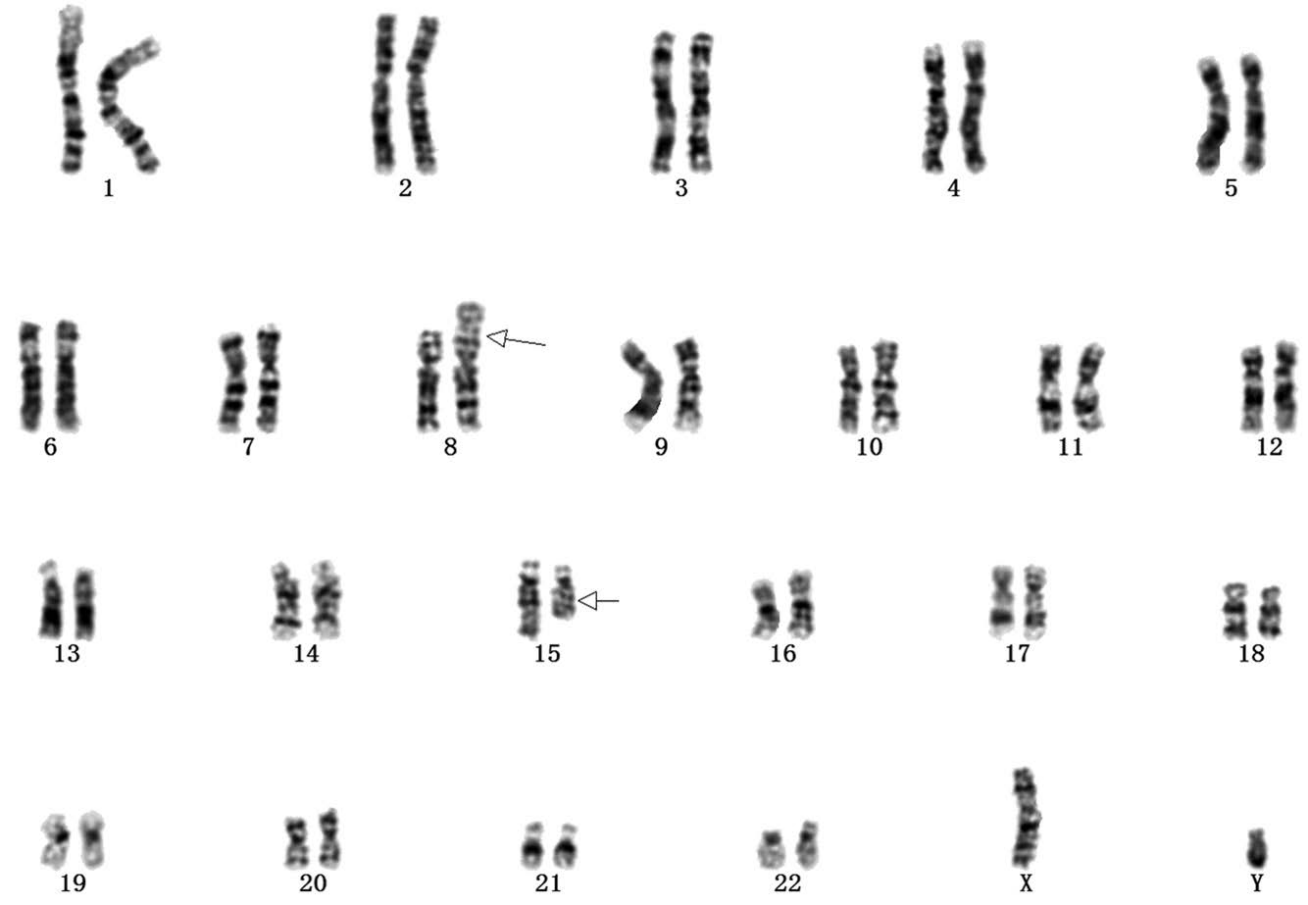
Genetic test results. Prenatal diagnosis of Chromosome Balanced Translocation in Case 1. Result of chromosome karyotype: The fetal chromosome karyotype was 46, XY, t (8; 15) (p23; q22).

A 31-year-old G2P1A0L0 pregnant woman presented for consultation, whose first child was delivered at full term by expected delivery but presented with multiple malformations, flexion, stiffness of the limbs, ectropion of the feet, and stenosis of the cardiac aorta, and survived for only 2 days after delivery. Ultrasound examination of the pregnant woman in the current pregnancy did not reveal any fetal structural abnormalities. However, because of her previous adverse reproductive history, she was advised to undergo amniocentesis and karyotyping to clarify the chromosomal status of the fetus. After amniocentesis, fetal karyotype analysis revealed that the fetal karyotype was 46, XY, t (8; 15) (p23; q22) (Fig. 1).

### 2.2. Case 2

**Figure 2.**
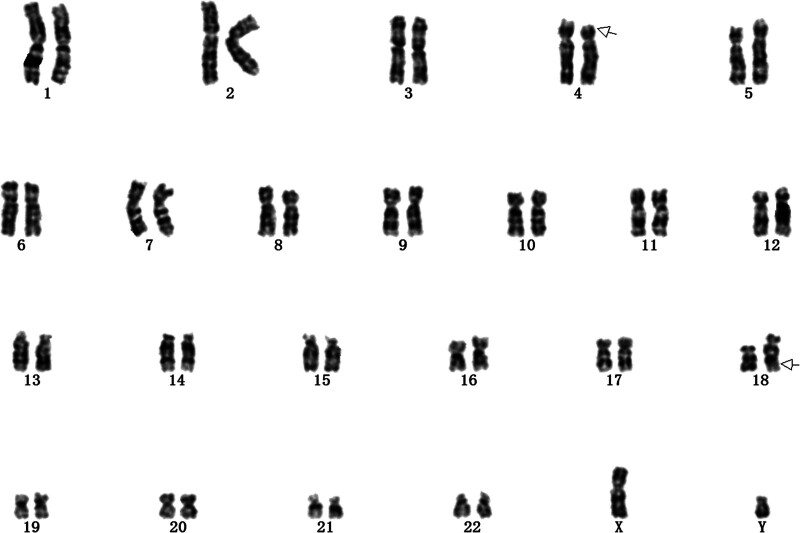
Genetic test results. Chromosome Balanced Translocation was diagnosed prenatally in Case 2. The chromosome karyotype result was: The karyotype of the fetal chromosome is 46,XY,t(4; 18)(p16; q23).

A 31-year-old G8P2A5L0 pregnant woman and her family presented for counseling. This pregnant woman had 4 previous spontaneous abortions, one induced abortion, and 2 regular full-term deliveries. Her first child was delivered with a congenital heart defect and lived for 6 months; while the second child was born with severe malnutrition, epilepsy, and clubfoot and lived for 9 months. The pregnant woman herself was mildly mentally disabled, unable to read or write, and unable to use electronic devices such as telephones. However, she had a fair memory and could retell what happened earlier. Due to her condition and history recurrent spontaneous abortions and history of malformed children, she was advised to undergo amniocentesis and fetal karyotyping to clarify the chromosomal status of the fetus, despite the low risk of trisomies 21, 18, and 13 on maternal serologic screening in the current pregnancy, and the fact that routine ultrasonography did not show any fetal anomalies. After amniocentesis, fetal karyotype analysis revealed that the fetal karyotype was 46, XY,t(4; 18)(p16; q23) (Fig. 2).

### 2.3. Case 3

**Figure 3.**
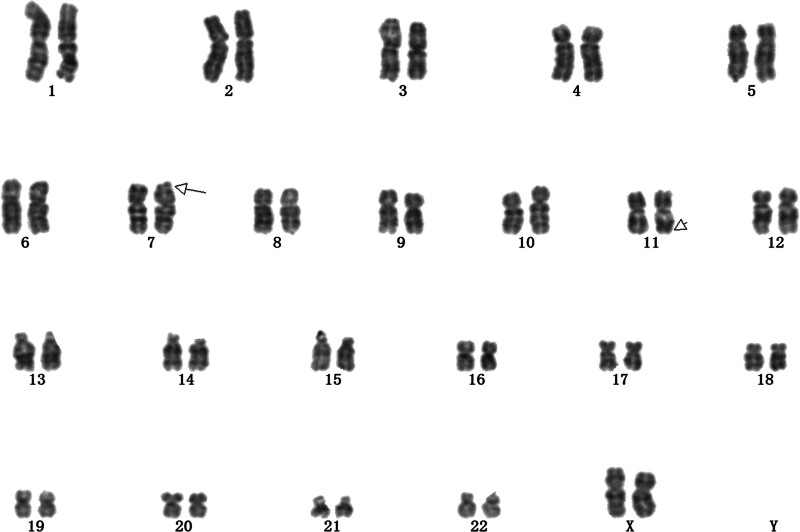
Genetic test results. Chromosome Balanced Translocation was diagnosed prenatally in Case 3. The chromosome karyotype result was: The karyotype of the fetal chromosome is 46,XX,t(7;11)(p22; q23).

A 33-year-old G1P0A0L0 pregnant woman with no fetal abnormalities on routine ultrasound and a high-risk mid-pregnancy serologic screen (trisomy 21 1/26) presented for consultation and was advised to undergo amniocentesis and fetal karyotyping to clarify the chromosomal status of the fetus. After amniocentesis, fetal karyotype analysis revealed that the fetal karyotype was 46, XX,t(7;11)(p22; q23) (Fig. 3).

All 3 couples had normal peripheral blood karyotypes, non-consanguineous marriages, no history of illness or medication use during pregnancy, no history of exposure to toxic or harmful chemicals or radiation, and no bad habits. After learning about the consequences of chromosomal abnormalities, all 3 pairs decided to continue their pregnancies and continued to undergo obstetric tests, including ultrasound, throughout their pregnancies. About a year after the delivery of their children, the staff of the Prenatal Diagnostic Center followed up with each of them by phone and been informed that the children physical development and intelligence were normal.

## 3. Discussion

Chromosomal translocations are abnormal chromosomal rearrangements that occur when segments are exchanged between 2 chromosomes, including reciprocal, rhodopsin, and insertion translocations. Chromosomal translocation is a complex process, and there are essential steps in the creation of a chromosomal translocation. First, a DNA double-strand break occurs at both loci, and then the ends of the broken pieces of DNA must be close to each other and illegitimately attached to each other.^[11]^

Separate breaks in 2 or more non-homologous chromosomes are called balanced chromosomal translocations. Broken segments are exchanged and rejoined, therefore there is no gain or loss of gene material during the translocation process. The broken points did not lead to the truncation of functional genes. The appearance of the carrier can be expected. However, during meiosis, carriers of chromosomal balanced translocations can generate a significant proportion of unbalanced gametes, putting the offspring at a much-increased risk of genetic problems, infertility, or repeated miscarriages.^[1,12,13]^In meiosis I, the translocated chromosome and its normal homologous chromosome can form a tetrad structure and segregate in a 2:2 3:1 or 4:0 pattern, where 2:2 segregation can be subdivided into para-segregation, neighbor-1 segregation, and neighbor-2 segregation. Gametes with regular genetic material can be formed only when the segments between the miter and the breakpoint undergo an odd number of interchanges and para- or neighbor-1 segregation; all other segregation patterns result in the production of chromosomally abnormal gametes.^[1,14]^

It has been suggested that the meiotic segregation pattern and the potential for generating imbalanced gametes are affected by the sex and age of the carrier, the location of the breakpoints, and the characteristics of the chromosomes involved in the rearrangements, Mutual translocations involving terminal breakpoints, for example, lead to a reduction in the number of embryos that are normal or carry balanced translocation chromosomes, and the proximal mitotic chromosomes involved in rearrangements are relatively more unstable than the central mitotic chromosomes, making it difficult to predict the probability that a carrier of a balanced chromosomal translocation will produce a normal or abnormal embryo, therefore, specific meiotic analyses should be performed for each specific translocation carrier to estimate genetic risk.^[14–18]^ Chromosomal translocations primarily cause chromosomal abnormalities in the embryo of female carriers but may also affect the process of spermatogenesis and fertility in male carriers. For example, men with X chromosome translocations are prone to complete sperm cessation.^[13,19]^

In both cases 1 and 2, female patients had a previous history of giving birth to malformed babies. The malformed newborns had difficulty surviving after birth or survived for shorter than 1 year, all of which met the criteria for undergoing prenatal diagnostic amniocentesis because most congenital disabilities, such as neural tube defects and congenital cardiac defects, occur in isolation and due to the interaction of multiple genes with environmental factors. Owing to the genetic component of such conditions, there is a tendency for such structural abnormalities to recur in the subsequent pregnancy in the family.^[6]^

In case 1, both spouses were phenotypically typical, and in order to exclude a history of previous malformed births due to the production of abnormal gametes by carriers of chromosomal translocations in both spouses, peripheral blood karyotyping was performed on both spouses in case 1. There were phenotypic abnormalities in the female patient in case 2, with a history of 2 malformed births and recurrent miscarriages; therefore, peripheral blood karyotyping was also performed in case 2 In case 3, both spouses have normal phenotypes, and the noninvasive prenatal diagnosis suggested an increased risk of trisomy 21. Therefore, an invasive prenatal diagnostic procedure was also performed, and peripheral blood karyotyping of both spouses was performed, considering the possibility of conceiving an abnormal fetus. The peripheral blood karyotypes of the couples in these 3 cases were expected, and all 3 cases of fetal chromosomal balanced translocations were considered primary fetal chromosomal balanced translocations.

## Author contributions

**Data curation:** Lan Yao, Yuxin Xia, Luyao Wang.

**Investigation:** Xueyu Zhao.

**Supervision:** Xun Kan, Yingli Lu.

**Writing – original draft:** Lan Yao.

**Writing – review & editing:** Lan Yao, Xun Kan, Yingli Lu.
